# From Targeting Somatic Mutations to Finding Inherited Cancer Predispositions: The Other Side of the Coin

**DOI:** 10.3390/diagnostics9030083

**Published:** 2019-07-26

**Authors:** Pascal Pujol, Thibault De La Motte Rouge, Frédérique Penault-Llorca

**Affiliations:** 1 Department of Cancer Genetics, University Hospital of Montpellier, 34000 Montpellier, France; 2 Department of Medical Oncology, Centre Eugene Marquis, 35000 Rennes, France; 3 Department of Pathology, Centre Jean Perrin, 63000 Clermont-Ferrand, France

**Keywords:** somatic analysis, cancer predisposing genes, secondary findings

## Abstract

The expanding use of tumor genome analysis by next generation sequencing to drive target therapies has led to increased germline findings in genes predisposing to hereditary cancer. These putative germline findings obtained from theranostic analyses, such as *BRCA1/2* gene testing, large panels, whole-exome, or whole-genome sequencing, need to be managed carefully and in an anticipated way with the patient. Before the genetic analysis of a tumor, specific information should be given to patients, who should be aware that the results may have extra-therapeutic medical issues for themselves and relatives. We previously published a list of 36 actionable genes predisposing to cancer for which informing the patient is recommended prior to pangenomic germline analysis because of available screening or preventive strategies. Here, we report clinical practice considerations and schemes for managing germline findings in tumor analyses, including written informed consent and a multidisciplinary approach involving an oncologist, molecular biologist/pathologist, and geneticist in case of germline findings. A somatic result showing a deleterious mutation in a known predisposing gene in a patient who has consented to this purpose should result in referral to a geneticist who is part of the multidisciplinary team. At any time of the somatic analysis process, the patient may have access to a geneticist consultation if additional information is required. This framework will optimally manage both personalized theranostic issues and specific preventive strategies for individuals and relatives; it will also simplify and accelerate the process of genetic testing.

## 1. Introduction

Personalizing treatment by using target therapies based on tumor somatic mutations has become a major endpoint in cancer care. It has already led to significant improvement in survival in both the metastatic and adjuvant setting, and next-generation tumor sequencing is increasingly being used for theranostic goals [1]. However, data from somatic mutation analyses such as large panels, whole-exome, or whole-genome sequencing may also reveal crucial germline findings for individuals and their families in terms of preventing heritable cancer. For instance, tumor exome analysis has led to a significant rate of germline cancer predisposition gene discoveries in individuals [2,3,4]. Therefore, this issue should be managed carefully and in an anticipated way with patients. Patients might be aware of such knowledge when they undergo somatic mutation tests, and the testing process must be clarified. 

In case of secondary findings, we previously reported a list of 36 actionable genes predisposing to cancer for which informing the patient is recommended because of available screening or preventive strategies [5]. In line with these recommendations, we propose a process for patients undergoing somatic tumor analysis that includes delivery of appropriate information, collection of informed consent, and a scheme for interactions among oncologist, molecular biologist/pathologist, and geneticist in a multidisciplinary approach. 

## 2. Subjects and Methods

In a previous work, the SFMPP (French society of predictive and personalized medicine) gathered 47 multidisciplinary experts and defined, from a primary list of 60 cancer-related genes, 36 “actionable” genes for which informing the clinician and the patient is recommended because of available screening or preventive strategies. Here we present the group’s additional considerations for somatic analysis. The group previously published written consent forms and an information media tool on secondary findings that could be used for this purpose [5,6]. 

## 3. Results

**Figure 1:**
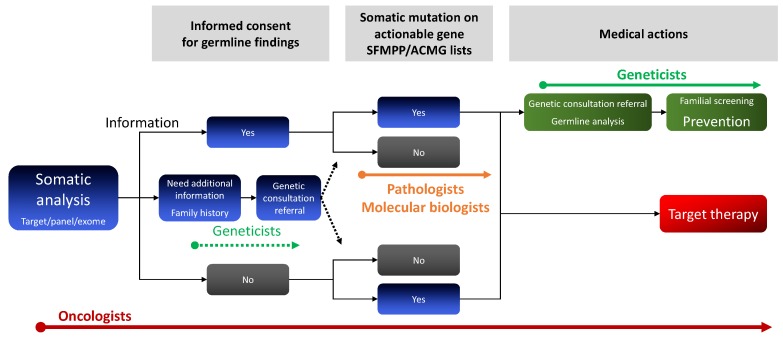
Multidisciplinary approach to managing putative germline findings of tumor analysis. NGS: next-generation sequencing. ACMG: American College of Medical Genetics.

At the time of somatic analysis, clinicians should give their patients clear information about the possibility and benefit of knowing a germline mutation for themselves and their relatives. A written informed consent for this issue should be obtained (Figure 1). After clear information is given, some patients may not want to address the germline issue and thus may only want access to the somatic information for a personalized therapeutic goal (Figure 1). Patients who require additional information on preventive strategies may be referred to geneticist consultations before the tumor test. In case of a tumor-detected mutation on actionable cancer genes listed by the American College of Medical Genetics or SFMPP [6,7], patients should be referred to a geneticist for constitutional analysis and specific counselling, support, and care. 

**Figure 2:**
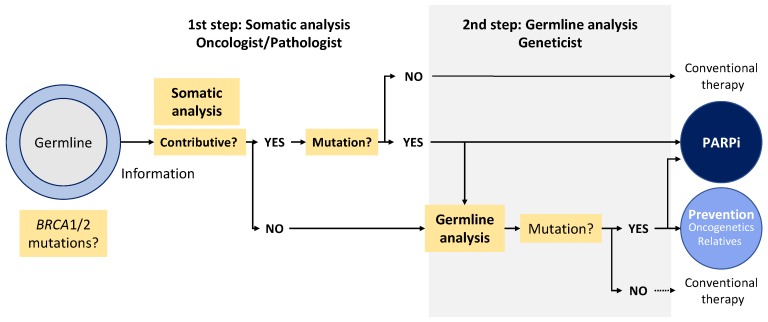
*BRCA1/2* testing for targeted therapies. PARPi: poly(ADP)ribose polymerase inhibitors. Not contributive: insufficient quantity of tumor cells or uninterpretable results.

The particular case of *BRCA1/2* somatic testing for targeted therapies is presented in Figure 2 and Table 1. We propose a model of a genetic testing pathway starting from tumor analysis based on oncologist information given to the patient before the test (Figure 2). Oncologists who received prior training on discussing the role of *BRCA* mutation (*BRCA*m) testing and genetic counseling techniques and/or genetic counselor members of a multidisciplinary team can provide patients with pre-*BRCA*m test counseling at the first step of somatic analysis (Figure 2). Patients who volunteer for the germline issue sign an informed consent. For some patients who do not want to deal with the germline issue after they are given information, the testing could be limited to the somatic level. If requested by the patient, additional pre-*BRCA*m test counseling would be provided by a geneticist or genetics counselor. After testing, the oncologist returns the results of the tumor analysis to the patient. For patients with positive *BRCA*m test results or negative *BRCA* test results but requiring additional counseling for familial risk, an appointment with a geneticist or a genetics counselor is recommended. In case of noncontributive tumor analysis (insufficient DNA quantity or quality, low percentage of cancer cells etc.), another biopsy, if available, could be sent to the pathologist, or a germline analysis could be proposed. 

## 4. Discussion

Tumor panels are now being used extensively by oncologists to identify clinically actionable mutations on cancer genes to drive target therapies. Because of the overlap between cancer genes involved at the somatic level and germline predisposition, patients might be aware of this possibility before undergoing a genetic tumor test. Here, we propose a model of a genetic testing pathway starting with somatic analysis by an oncologist. The model includes information tools [6], collection of informed consent, and possibility of referral to a specialized consultation of oncogenetics in case of positive results and/or additional information the patient requires before the test. 

Recently, results of the ENGAGE study demonstrated that an oncologist-led testing process for mutated *BRCA1* or *2* genes is feasible in ovarian cancer; the oncology-led testing pathway reported was associated with high levels of acceptance and satisfaction among patients with ovarian cancer [8].

National guidelines recommend that *BRCA*m testing be provided to all patients with a diagnosis of epithelial ovarian cancer [9,10]. This recommendation was initially proposed because of the high prevalence of *BRCA*m findings in patients diagnosed with ovarian cancer [11]. In addition, in Europe, these guidelines include a search for *BRCA1/2* tumor mutations because they are European Medicines Agency-approved targets for treatment with olaparib for ovarian cancer patients with platinum-sensitive relapse [12]. Hence, somatic mutations may be considered the first endpoint for this targeted therapy. However, in 70% to 80% of cases, the finding of a somatic mutation leads to the discovery of a germline mutation, with individual- and family-important issues in terms of screening and prevention [11]. Of note, recent evidence shows that only 54% of women with a *BRCA*m have a family history of breast and/or ovarian cancer [8]. Thus, family history cannot be considered a sufficient predictor of the need for information on putative germline mutation.

With the improvement in sequencing technologies and bioinformatics tools, we can now search for the largest genomic rearrangement from tumor analysis [13,14]. Thus, tumor analysis may become the preferred method to detect both exclusive somatic mutations and germline mutations, including copy number variations/large genomic rearrangements. The somatic analysis process will diminish the total number of genetic analyses to perform, because a negative test result will avoid constitutional analysis in most cases of contributive samples.

In some cases, patients may not want knowledge of a germline predisposition. We and others showed that only some relatives who are at risk of carrying a familial *BRCA1/2* or mismatch repair mutation within a family undergo genetic testing [15,16]. Thus, respecting the autonomy of patients not to have access to the germline information should preserve the possibility of the patient opting for somatic analysis only.

One important practical issue is the limited availability of genetics counselors. Also, the delay for patients with advanced ovarian and breast cancer accessing the geneticist consultation and germline *BRCA*m testing may not be compatible with the timing of personalized therapy [17,18]. For example, the average wait time for a genetics appointment is about 14 weeks in the United Kingdom and France [18,19]. A new, more streamlined testing approach is needed to shorten testing turnaround times. Because many women with advanced ovarian and breast cancer may not be concerned with the distressful issue of the hereditary risk, tumor-led analysis may favor a better use of geneticist consultation resources.

At this time, genetic counseling is recommended both when genetic testing is offered to the patient and after genetic test results are disclosed [11,18]. In case of satisfactory information given by the clinician performing the initial genetic testing, we propose that only patients with a positive test result be referred to a geneticist, as previously suggested [8,20]. We believe that a more streamlined testing approach, potentially starting from somatic analysis, with information given to the patient by the oncologist, could shorten testing turnaround times but preserve the autonomy of patient for access to a germline finding.

## Figures and Tables

**Table 1:** Arguments for proposing a first-line somatic process for theranostic *BRCA1/2* testing.

Arguments in Favor of Starting with Somatic Analysis
Need to detect exclusive somatic mutation (5–7% ovarian cancer, breast?)
Possibility to detect large genomic rearrangement by somatic next-generation sequencing
Emergency of the results for therapeutic purpose
Cost (2 analyses systematically performed if starting by germline)
Oncologist more aware of BRCA issues and in first line
Most patients negative for BRCA mutation will not need genetic counselling

## Abbreviations

NGS	next-generation sequencing
ACMG	American College of Medical Genetics
SFMPP	Société française de médecine prédictive et personnalisée
